# Assessment of Bio-Compounds Content, Antioxidant Activity, and Neuroprotective Effect of Red Cabbage (*Brassica oleracea* var. *Capitata rubra*) Processed by Convective Drying at Different Temperatures

**DOI:** 10.3390/antiox12091789

**Published:** 2023-09-21

**Authors:** Antonio Vega-Galvez, Luis S. Gomez-Perez, Francisca Zepeda, René L. Vidal, Felipe Grunenwald, Nicol Mejías, Alexis Pasten, Michael Araya, Kong Shun Ah-Hen

**Affiliations:** 1 Departamento de Ingeniería en Alimentos, Universidad de La Serena, Avda. Raúl Bitrán 1305, La Serena 1700000, Chile; 2 Facultad de Medicina, Instituto de Neurociencia Biomédica (BNI), Universidad de Chile, Santiago 8380000, Chile; 3 Centro FONDAP de Gerociencia, Salud Mental y Metabolismo (GERO), Santiago 8380000, Chile; 4 Centro de Biología Integrativa, Facultad de Ciencias, Universidad Mayor, Santiago 8380000, Chile; 5 Centro de Investigación y Desarrollo Tecnológico en Algas (CIDTA), Facultad de Ciencias del Mar, Universidad Católica del Norte, Larrondo 1281, Coquimbo 1780000, Chile; 6 Facultad de Ciencias Agrarias y Alimentarias, Instituto de Ciencia y Tecnología de los Alimentos, Universidad Austral de Chile, Valdivia 5090000, Chile

**Keywords:** antioxidant activity, bio-compounds, cytotoxicity, neuroprotective effects, red cabbage

## Abstract

Parkinson’s disease (PD) is the second most common neurodegenerative disorder, and no efficient therapy able to cure or slow down PD is available. In this study, dehydrated red cabbage was evaluated as a novel source of bio-compounds with neuroprotective capacity. Convective drying was carried out at different temperatures. Total phenolics (TPC), flavonoids (TFC), anthocyanins (TAC), and glucosinolates (TGC) were determined using spectrophotometry, amino acid profile by LC-DAD and fatty acid profile by GC-FID. Phenolic characterization was determined by liquid chromatography-high-resolution mass spectrometry. Cytotoxicity and neuroprotection assays were evaluated in SH-SY5Y human cells, observing the effect on preformed fibrils of α-synuclein. Drying kinetic confirmed a shorter processing time with temperature increase. A high concentration of bio-compounds was observed, especially at 90 °C, with TPC = 1544.04 ± 11.4 mg GAE/100 g, TFC = 690.87 ± 4.0 mg QE/100 g and TGC = 5244.9 ± 260.2 µmol SngE/100 g. TAC degraded with temperature. Glutamic acid and arginine were predominant. Fatty acid profiles were relatively stable and were found to be mostly C18:3n3. The neochlorogenic acid was predominant. The extracts had no cytotoxicity and showed a neuroprotective effect at 24 h testing, which can extend in some cases to 48 h. The present findings underpin the use of red cabbage as a functional food ingredient.

## 1. Introduction

Red cabbage (*Brassica oleracea* var. *Capitata rubra*) has its origin in Europe, but it is cultivated today all over the world. It is recognized by consumers to be highly nutritive, being a rich source of micronutrients and phytochemicals, abundant in fibers, vitamins, polyphenols (including flavonoids, and especially anthocyanin), and glucosinolates that are secondary plant metabolites with positive effects on human health [1,2]. As proved by epidemiological studies, consumption of foods rich in glucosinolates and antioxidant components [3], which is the case of red cabbage, is linked to a reduced risk of hepatic steatosis, cancer, and cardiovascular disease. Red cabbage has strong antioxidant capacity and health-promoting properties due to its nutritional components comprising anthocyanins, phenolic acid derivatives, flavonoids, vitamins, glucosinolates, and isothiocyanates [4,5]. Release of compounds such as 4-(methylsulfinyl)butyl ITC (4MSOB-ITC, sulforaphane) from red cabbage [6] has been reported to have strong chemopreventive properties [7]. Oral intake of cabbage extracts has also been found to reduce the oxidative stress in the livers and hearts of rats [8] when ingested in a dose of around 100 mg/kg body weight. Zielinska et al. [9] also reported significant anti-inflammatory effects in assays with mice suffering intestinal damage through Crohn’s disease or ulcerative colitis. 

Parkinson’s disease (PD) is the second most common neurodegenerative disorder; it is chronic and progressive and results primarily from the death of nerve cells in the substantia nigra, a region of the midbrain where dopamine is produced, which leads to a dopamine deficit. PD is also characterized by the accumulation of protein aggregates, consisting mainly of α-synuclein [10]. An efficient therapy able to cure or slow down PD is not available. However, it has been hypothesized that oxidative stress and reactive oxygen species (ROS) could be the prime causes of neurodegeneration in PD, which could be the basis for neuroprotection therapy [11]. In this sense, some studies have shown the connection between antioxidant potential from phytochemicals and neuroprotection capacity, associating dietary antioxidants with a lower risk of PD [12]. Hence, phytochemicals, in particular flavonoids that have antioxidant properties, could be applied in the treatment of neurological disorders induced by oxidative stress [13,14]. Guo et al. [15] have investigated the effect of polyphenols in green tea extract on cell viability using SH-SY5Y cells and the neurotoxin 6-hydroxydopamine (6-OHDA). Similarly, Cho et al. [16], using SH-SY5Y cells, investigated the effect of green tea extract on the pathogenesis of PD and reported positive effects on cell viability. The major active constituents of turmeric have also been investigated by Jaisin et al. [17], who reported a significant increase in cell viability when the cells were incubated with curcumin for 30 min prior to the addition of 6-OHDA. The cytotoxic effects of curcumin at concentrations above 10 mmol/L are also considered to be responsible for the reported anti-cancer effects. Curcumin apparently reduces ROS production induced by neurotoxin, showing neuroprotective effects [18]. It has also been reported that cabbage extracts have the ability to inhibit lipid peroxidation in brain tissue, giving it a neuroprotective potential [19]. Ghareaghajlou et al. [20] reported that anthocyanins present in red cabbage have an important role in the control of neuroinflammation and oxidative stress, contributing to neuronal cell protection. On the other hand, compounds such as polyunsaturated fatty acids (PUFAs) exert anti-inflammatory and antioxidant activity and may be promising in delaying or preventing PD by attenuating neuroinflammation [21]. Since diet has shown some effectiveness in improving symptoms, this study proposes an evaluation of extracts from dehydrated red cabbage as a novel source of bioactive compounds with neuroprotective capacity.

Red cabbage is commonly consumed as fresh-cut salads or cooked vegetable dishes, but it is also, in its dehydrated state, an important versatile raw material with multiple applications in the food industry, for instance, as an ingredient in soups or other food products. Its content of anthocyanins and other bioactive compounds [22] contributes to its health-promoting properties, so the effects of drying should be well-calibrated to maintain the related nutritional health benefits of the dried products [8]. Hot air drying is a conventionally used dehydration process well known for being readily available and easily implemented [23]. However, the degradation of thermolabile bioactive constituents is quite common, and for red cabbage, little is known about the effect of hot air temperatures in a convective drying process on the actual state of the bioactive compounds and their health-related properties. So far, reports on these aspects have scarcely or altogether not been published. Therefore, the research proposal consists of evaluating the effects of different levels of hot air-drying temperatures from 50 to 90 °C at 10 °C intervals, quantifying the contents of bioactive compounds such as total polyphenolics, total flavonoids, total anthocyanins, total glucosinolates, antioxidant capacity through DPPH and ORAC assays as well as determining the phenolic, amino acid and fatty acids profiles, along with cytotoxicity and neuroprotection assays with SH-SY5Y human cells.

## 2. Materials and Methods

### 2.1. Solvents and Reagents

All reagents used were of analytical or HPLC grade and were purchased from SIGMA Aldrich (St. Louis, MO, USA): methanol (MeOH), (0.1%) formic acid, dimethyl sulfoxide (DMSO), Folin–Ciocalteu reagent, (20%) sodium carbonate solution (Na_2_CO_3_), (5%) sodium nitrite solution (NaNO_2_), (10%) aluminum trichloride solution (AlCl_3_), sodium hydroxide (NaOH), sodium tetrachloropalladate II (Na_2_PdCl_4_) reagent, Sinigrin (Sng), salicylic acid, acetonitrile (C_2_H_3_N), hydrochloric acid (HCl), borate buffer solution, o-phthalaldehyde (OPA), acetonitrile, boron trifluoride (BF_3_), Sodium chloride (NaCl), 6-Hydroxy-2,5,7,8-tetramethylchoman-2-carboxylic acid (Trolox), 2,2-diphenyl-1-picrylhydrazyl (DPPH), 2,2-azobis(2-amidinopropane) dihydrochloride (AAPH), gallic acid, quercetin, Triton X100, Sytox green, Dulbecco’s modified Eagles medium (DMEM), fetal bovine serum (FBS) and Nitrogen (>99.98%). 

### 2.2. Raw Material and Drying Conditions

Fresh red cabbage (*Brassica oleracea* var. *Capitata f. rubra*) was purchased from a greengrocer in Coquimbo, Chile. The cruciferous was washed after removing any visibly spoiled leaf and chopped into pieces of 1 cm width. The samples underwent a blanching process according to Tao et al. [24] with some modifications; the sample was immersed in boiled water for 30 s, rapidly cooled in ice water, and allowed to drip in a sieve. For the drying process at 50, 60, 70, 80, and 90 °C with an air velocity of 1.5 m/s, samples of 30 ± 1 g of the blanched red cabbage were spread in metal baskets as a thin layer of 1 cm and placed in a convective hot air dryer. The weight loss was recorded at different time intervals on a digital balance (Radwag AS 220-R2, Torunska, Poland) until constant weight. The drying kinetics was carried out in triplicate, and the moisture ratio (*MR*, dimensionless) was determined according to Equation (1) [25].

(1) $$MR=\frac{X_{wt}-X_{we}}{X_{w0}-X_{we}}$$

where *X_w_*_0_, *X_wt_*, *X_we_* (g water/g d.m. (dry matter)) are respectively water content at the start of the drying process, after time *t*, and at the final equilibrium state.

### 2.3. Determination of Bioactive Compounds 

To determine the total content of bioactive compounds and their antioxidant activity, extracts of the fresh and the dehydrated red cabbage were prepared with 80% aqueous methanol in a ratio of 1:2 and 1:10, respectively, as described by Ke et al. [26]. The mixture was agitated on an orbital shaker at 250 rpm for 1 h. Subsequently, it was centrifuged at 5000 rpm for 10 min at 4 °C. The supernatant was filtered and recovered. The solid residue was used for a new extraction process that was repeated thrice. The three combined supernatants were evaporated to remove solvent and lyophilized. For reconstitution of the extract, 5 mL of methanol/formic solution (99:1) was used. 

The extracts used for the neuroprotection assays were obtained in a similar way but in proportions of 1:2 for the fresh and 1:4 for the dehydrated red cabbage and reconstituted in DMSO.

#### 2.3.1. Total Polyphenolics, Total Flavonoids, Total Anthocyanins and Total Glucosinolates

The total polyphenolic content (TPC) was determined using a spectrophotometric method as described by Uribe et al. [27] but with some modifications. A 0.5 mL aliquot of the red cabbage extract was mixed with 0.5 mL of Folin–Ciocalteu reagent and incubated for 5 min in the dark. Afterward, 2 mL Na_2_CO_3_ solution (200 mg/mL) was added, and the mixture was left to react for 15 min before adding 10 mL of distilled water and centrifugation for 5 min at 5000 rpm (5804 R, Eppendorf, Hamburg, Germany). Absorbance was then measured at 725 nm using a spectrophotometer (Spectronic 20 Genesys^TM^, Chicago, IL, USA). Gallic acid was the reference standard to obtain the calibration curve (*y* = 0.0037*x* + 0.002; R^2^ = 0.9965). TPC was expressed as mg gallic acid equivalent (GAE)/100 g d.m.

Total flavonoid content (TFC) was determined according to the method described by Dini et al. [28]. 0.5 mL of red cabbage extract was mixed with 2 mL of distilled water. The reaction was started by adding 0.15 mL of aqueous NaNO_2_ solution at 5%. The mixture was then incubated in the dark for 5 min before adding 0.15 mL of aqueous AlCl_3_ solution at 10%, leaving it in the dark for a further 6 min. Finally, 1 mL NaOH (1 M) and 1.2 mL of distilled water were added. The absorbance was measured at 415 nm. Quercetin was the reference standard to obtain the calibration curve (*y* = 0.0017*x* − 0.0035; R^2^ = 0.9966). TFC was expressed in mg of quercetin equivalents (QE)/100 g d.m.

The total anthocyanins content (TAC) was estimated using the pH differential method [29]. The red cabbage extract was diluted with pH 1.0 and pH 4.5 buffers. The absorbance was measured at 510 nm and 700 nm for each of the buffers. TAC (expressed in terms of cyanidin-3-glucoside) was calculated using the following equations:

(2) $$A=\left(A_{510}-A_{700}\right){pH}_{1.0}-\left(A_{510}-A_{700}\right){pH}_{4.5}$$

(3) $$TAC=(A\times MW\times DF\times V_{e}\times 1000)/(\varepsilon \times 1\times M\;)$$

where MW is the molecular weight of cyanidin-3-glucoside (449 g/mol), DF is the dilution factor, $V_{e}$ is the volume of extract, ε is the molar extinction coefficient of cyanidin-3-glucoside (26,900), and M is the mass of extracted red cabbage.

Total glucosinolates content (TGC) was determined as described by Aghajanzadeh et al. [30], with some modifications. 60 μL of red cabbage extract and 1800 μL of 2 mM Na_2_PdCl_4_ were mixed, shaken, and incubated for 30 min in the dark. The absorbance was measured at 450 nm. Sinigrin was the reference standard to obtain the calibration curve (*y* = 0.084*x* − 0.0286, R^2^ = 0.9983). The results were expressed as the equivalent of sinigrin in 100 g of sample (μmol SngE/100 g d.m.).

#### 2.3.2. Extraction, Identification and Quantification of Phenolic Compounds 

200 mg de red cabbage powder for each treatment was extracted with 990 µL of 80% (*v*/*v*) methanol: water with the internal standard added (10 µL salicylic acid 1000 µg/mL) into the extraction solvent during the extraction. After the addition of the solvent, the mixture was stirred in a cellular disruptor three times for 3 min each time. After extraction, the mixture was centrifuged at 12,000 rpm for 15 min to collect the supernatant. The supernatant was filtered through a 0.22 µm PTFE membrane, and 200 µL was dried with nitrogen in a BIOBASE sample concentrator MD200-1. The residue was resuspended in 50 µL of extraction solution and introduced in a vial insert for chromatographic analysis. Detection of phenolic compounds in the extracts was carried out by LC-HRMS analyses. The instrumental analysis was developed using a Dionex Ultimate 3000 UHPLC system (Thermo Fisher Scientific, Sunnyvale, CA, USA). A reversed-phase HPLC column Kinetex^®^ C18 (50 mm × 2.1 mm; 2.6 µm), both from Phenomenex (Torrance, CA, USA), was used. The flow rate was set at 0.4 mL min^−1^, and the injection volume was 5 µL. The mobile phase was used in gradient mode as follows: 80% of eluent A (100% water containing 48 mM formic acid (FA)) and 20% of eluent B (50% acetonitrile:50% water) were maintained with 48 mM FA for 1.0 min, followed by a linear increase to 100% B in 8.0 min and holding for 5 min. After, add eluent C (methanol) for 2 min and, finally, equilibrate with the initial conditions for 2 min. The detection of phenolic compounds was carried out by a high-resolution mass spectrometer Q Exactive Focus with Orbitrap detector equipped with an electrospray interphase HESI II (Thermo Fisher Scientific, Sunnyvale, CA, USA). The HESI was operated in negative ionization mode with a spray voltage of 2.5 kV. The temperature of the ion transfer tube and the HESI vaporizer were set at 320 °C. Nitrogen (>99.98%) was employed as sheath gas and auxiliary gas at pressures of 20 arbitrary units. The data were acquired in Full MS and data-dependent (ddMS2) acquisition mode. The mass scan range was set at 100 to 800 *m*/*z* with a mass resolution of 70,000, the automatic gain control (AGC) was established at 5 × 10^4^, and the maximum injection time (IT) was 3000 ms. For ddms2, the mass resolution was set at 70,000, AGC at 5 × 10^4^, and IT at 3000 ms. In both cases, the isolation windows were 2 *m*/*z*. For identification, the compound was searched in the PubChem database. The exact mass was calculated in negative mode using the monoisotopic mass of the compound (theoretical mass). Then, it was compared with the experimental exact mass, and the error was calculated. Due to their low concentration, some compounds could not be fragmented. For quantification, the internal standard (IS) method was used, and the relative concentration was calculated in relation to the salicylic acid internal standard according to Equation (4). The final concentration is calculated considering the initial dilution factor.

(4) $$\left[\mathrm{Analyte}\right]=\left[\mathrm{IS}\right]\times \frac{\mathrm{area}\mathrm{analyte}}{\mathrm{area}\mathrm{IS}}$$

#### 2.3.3. Amino Acid and Fatty Acid Profiles 

The amino acid profile was determined using the HPLC pre-column derivatization method [31]. To a semi-capped hydrolysis tube containing 200 mg of the sample, 10 mL of 6 M HCl was added, and the mixture was incubated for 24 h at 120 °C. After hydrolysis, the solution was transferred to a 50 mL volumetric flask that was filled up to the mark with distilled water. Then, 100 μL aliquot of the solution was adjusted to pH 10.0 with 1.0 mL borate buffer and concentrated to dryness in a rotary evaporator. The concentrated extracts were reconstituted to a volume of 200 μL with borate buffer (pH 10.0) and filtered using a 0.22 μm Nylon syringe filter for its derivatization. Pre-column derivatization of the amino acids was performed using the autosampler Jasco AS-2055 programming. The amino acids were derivatized with OPA and detected using a ZORBAX Eclipse AAA amino analysis column (3.5 μm, 4.6 × 150 mm) and an Agilent HPLC system (Santa Clara, CA, USA) regulated at 40 °C. The mobile phase was composed of borate buffer (pH 7.8; A), acetonitrile–methanol–water (90:90:10, *v*/*v*/*v*; B), and 100% methanol (C) at a flow rate of 2 mL/min. The following gradient was used for the elution procedure: 0–1.9 min, 100% (A); 18.1–18.6 min, 42% (A) 58% (B); 22.3 min, 30% (A) 70% (B); 22.40–26.00 min, 100% (C); 26.10–28.00 min, 100% (A). The detection was performed by recording the spectra between 240 nm and 400 nm. The measurement was made at 338 nm.

The fatty acid profile was determined as described by Folch et al. [32], using 1.000 ± 0.005 g d.m. of sample for the extraction process, followed by the subsequent conversion of the lipids into fatty acid methyl esters (FAMEs), employing boron trifluoride and 14% aqueous methanol (BF_3_-MeOH) [33]. FAMEs were extracted with hexane, followed by washing with 20% aqueous NaCl. The organic fraction was recovered and evaporated to dryness. The extract was reconstituted in 1 mL hexane. The FAMEs were quantified by gas chromatography (Clarus 600 FID model, PerkinElmer, Waltham, MA, USA) equipped with a flame ionization detector (GC-FID) and Omega Wax 320 capillary column (30 m × 0.320 mm × 0.25 μm, Supelco, Bellefonte, PA, USA) with temperature limits of 20–250 °C. A temperature ramp of 60 °C was maintained for 3 min and was further increased at 10 °C/min up to 260 °C. Nitrogen was used as carrier gas at a flow rate of 1.0 mL/min. Individual fatty acid was quantified by comparing retention times and peak areas with the FAME standard (Supelco 37 Component FAME Mix, Sigma, n° CRM47885, St. Louis, MO, USA).

### 2.4. Antioxidant Activity-DPPH and ORAC Assays

The antioxidant activity of red cabbage was determined using the DPPH (2,2-diphenyl-2-picryl-hydrazyl) assay developed by Brand-Williams et al. [34]. 100 µL of red cabbage extract was added to 3.9 mL of DPPH solution (50 µM in methanol) and left to react in the dark for 30 min. Subsequently, absorbance was measured at 517 nm. Trolox was used as the reference standard for the calibration curve (*y* = −0.4688*x* + 0.4052, R^2^ = 0.9987). The results were expressed as µmol TE (Trolox equivalent)/100 g d.m.

The antioxidant activity of red cabbage was also determined using the ORAC assay, carried out according to Uribe et al. [35]. 50 μL of red cabbage extract was mixed with 40 μL of phosphate buffer (pH 7.4) in a 96-well multiplate reader (Perkin Elmer, Victor X3, Hamburg, Germany). 200 μL of fluorescein solution was added to each well and incubated for 20 min at 37 °C. Subsequently, 35 μL of a 0.36 M solution of AAPH were added to each well for determination at excitation and emission wavelengths of *λ*_ex_ of 485 nm and *λ*_em_ of 535 nm, respectively. The calibration curve for the ORAC assay was obtained by plotting Trolox concentrations between 5 and 250 μM versus the area under the fluorescence decay curve, obtaining the following equation: *y* = −0.0012*x* + 0.5901 (R^2^ = 0.9808). The results were expressed as µmol TE (trolox equivalent)/100 g d.m. 

### 2.5. Neuroprotective Potential

The neuroprotective effect was determined using the Parkinson’s disease (PD) model based on alpha synuclein-preformed fibril (α–syn PFF) treatment [36]. SH-SY5Y cell lines derived from human neuroblastoma were seeded in a 96-well plate at 1 × 10^4^ cells per well in 100 µL of medium (DMEM + 10% FBS). SH-SY5Y cells were treated with three different concentrations (10, 50, and 100 μg/mL) of red cabbage extracts. Triton X100 was used as a positive control, and DMSO as a negative control. After 24 h, the cytotoxicity was determined by measuring the Sytox green intracellular levels. 

To evaluate the cellular neuroprotective effect of red cabbage extract, SHSY5Y cells were seeded in a 96-well plate at 1 × 10^4^ cells per well in 100 µL of medium (DMEM + 10% FBS). The cells were treated with alpha synuclein-preformed fibril (α–syn PFF, 1 µM/mL) to trigger cytotoxicity as a PD model. Red cabbage extracts (fresh, 50, 60, 70, 80, and 90 °C) were added together with α–syn PFF at a concentration of 100 µg/mL. After 24 or 48 h, the cytotoxicity was determined by measuring the Sytox green levels.

### 2.6. Statistical Analysis

The results were expressed as means ± standard deviation (SD) and statistically analyzed using the RStudio software (V. 1.4.1717). The means were compared by an analysis of variance (ANOVA) and Tukey’s test to estimate the significance among the main effects at the 5% probability level. Pearson’s correlation coefficients and principal component analysis (PCA) were performed to assess the relationship between the results.

## 3. Results

### 3.1. Drying Characteristics of Red Cabbage in Hot Air at Different Temperatures

**Figure 1:**
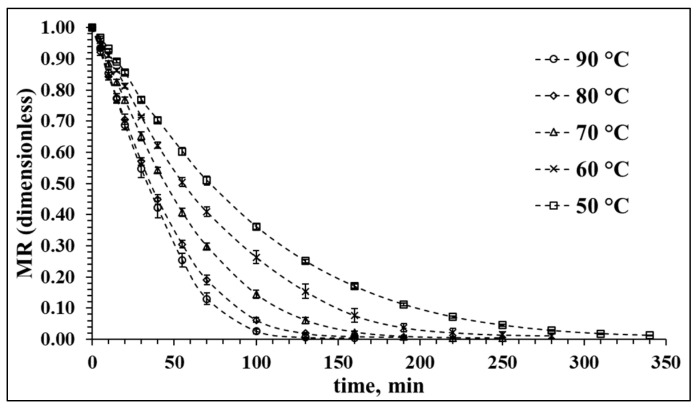
Drying kinetics of red cabbage at different process temperatures. *MR*: Moisture ratio (dimensionless).

The drying characteristic of the red cabbage, as shown in Figure 1, is a function of the hot air temperature. In this study, the hot air-drying process was conducted at several temperatures between 50 and 90 °C at regular intervals of 10 °C. The positive effects of drying temperature on mass transfer are reflected in the resulting drying time. The moisture ratio (*MR*) during the dehydration of the red cabbage samples decreased more rapidly as the drying air temperature increased. The whole drying process took place mainly in the falling rate period, during which the mechanism of mass transfer is predominantly internal molecular diffusion [37]. An increase in drying temperature led to a decrease in the time required to achieve equilibrium moisture content at an *MR* value of around 0.02; this *MR* value was achieved after circa 100 min at 90 °C, while at 50 °C, almost 340 min was required. Although drying at 90 °C is energetically more efficient, effects on quality parameters may not necessarily be more convenient, so the analysis of different quality aspects is of utmost importance to assess the hot air-drying process of red cabbage.

### 3.2. Bioactive Compounds

**Figure 2:**
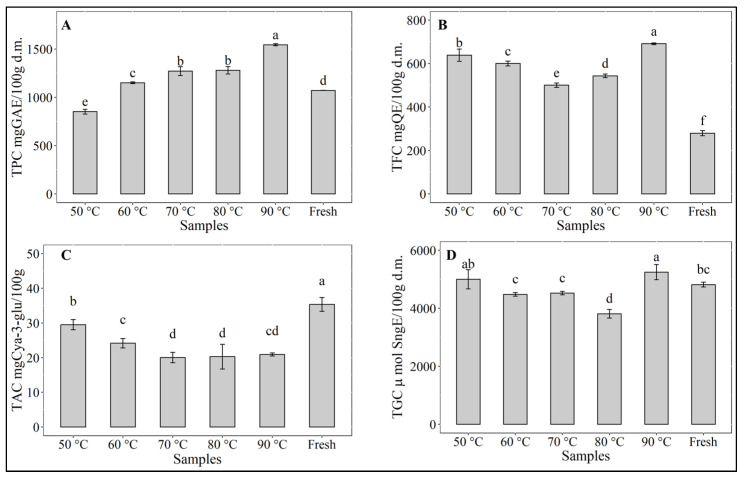
Bio-compounds from dried and fresh red cabbage extracts. (**A**) TPC (total phenolic content), (**B**) TFC (total flavonoid content), (**C**) TAC (total anthocyanin content), and (**D**) TGC (total glucosinolate content). Different letters indicate significant differences (*p* > 0.05).

As can be seen in Figure 2, the drying process caused significant changes (*p* < 0.05) in the contents of all bioactive compounds. Interestingly, total polyphenolic content (Figure 2A) increased significantly (*p* < 0.05) at drying temperatures over 60 °C with a noticeable highest value at 90 °C, while at 50 °C a significant (*p* < 0.05) decrease was observed. No significant difference (*p* > 0.05) was observed between the TPC of the samples dried at 70 and 80 °C. The TPC of the sample dried at 60 °C is slightly but significantly (*p* < 0.05) higher than the TPC of the fresh sample. Similar observations have been reported for myrtle leaves [38] or for banana peels [39], where the findings were related to longer exposure to heat at lower drying temperatures. The loss of polyphenolics occurring at 50 °C can be attributed to the relative instability of these compounds during prolonged thermal treatment [40] as well as to binding with proteins or changes in the chemical structure [41]. On the other hand, the increase in TPC at temperatures from 60 to 90 °C has been reported in the literature and was related to the availability of precursors arising from non-enzymatic interconversion between phenolic molecules [42].

Similarly, the total flavonoid content (Figure 2B) was also significantly higher (*p* < 0.05) than the TFC of the fresh samples. The highest level of TFC also occurred during drying at 90 °C. It is interesting to note that the TFC of the sample dried at 50 °C was the second highest content. As reported by Liu et al. [43], a significant increase in the content of flavones, flavonols, and flavonoid polymers was observed in the withering stage during tea leaves (*Camelia sinensis*) processing, which was related to increased enzyme activities. This may explain the noticeably high level of flavonoids found in samples dried at 50 °C, where the potential of the existing enzymes in the red cabbage was not inactivated, as it occurred at higher drying temperatures. It was possible for the enzymes to be active during the prolonged drying time and to contribute to the increase in flavonoids. At temperatures between 60 and 80 °C, the length of drying time probably affected the formation of flavonoids, which is not the case for drying at 90 °C. Geng et al. [44] have also reported an increase in specific flavonoids, including kaempferol, isorhamnetin, and quercetin, during hot air-drying of sea buckthorn (*Hippophae rhamnoides* L.) down to a moisture content below 10%, so that in general, the drying process may be considered favorable to enhance the quality of dehydrated red cabbage with respect to flavonoids content. Recently, eriodictyol-7-O-rutinoside-4′-O-sophoroside, belonging to the class of dihydroflavone, has been reported as a major flavonoid component in red cabbage [45]. As a flavonoid glycoside, similar to baicalin, it may show antitumor and antioxidant effects [46]. Many research works have also shown flavonoids’ ability to scavenge free radicals, as well as to improve glycemic control, lipid profile, and antioxidant status [47]. Therefore, due to its potential benefit to health, the flavonoid content may be a useful parameter in composing healthy human diets [48].

Red cabbage is well-known as an excellent source of anthocyanins [49]. However, the content of anthocyanin depends greatly on variety, maturation stage, or agricultural practices [50]. Consumption of anthocyanins has been related to numerous health benefits [51]. The acylated and non-acylated cyanidin glycosides of red cabbage anthocyanins have been reported to have excellent stability during heating in a pH range between 3 and 7 [52,53]. In this study, the anthocyanins of the red cabbage samples (Figure 2C) suffered without exception degradation during hot air-drying at temperatures between 50 and 90 °C. However, drying at 50 °C seemed to be more favorable to the stability of the anthocyanins. TAC at 50 °C was reduced by about 15% and showed a significant difference (*p* < 0.05) to TAC in the samples obtained during drying at the other temperatures, where the differences in TAC of samples dried at 70, 80, and 90 °C were not significant (*p* > 0.05). A loss of anthocyanins of about 40% was observed in the latter cases. Liu et al. [54] found that increasing temperature to 60 °C led to the formation of chalcone and consequent color fading of the anthocyanins. Jampani and Raghavarao [55] reported a reduction of 23% in the content of anthocyanins at 80 °C, while Ekici et al. [56] found a loss of red cabbage anthocyanin of only 2.57% during heating for 120 min at 70 °C, attributing this high heat stability of the red cabbage anthocyanins to the acylated anthocyanin compositions. Similarly, Dyrby et al. [53] argued that red cabbage anthocyanins had higher thermal stability than those in blackcurrant, grape skin, and elderberry due to the complex sugar residues of red cabbage anthocyanins. 

Total glucosinolates content (Figure 2D) showed, with respect to the fresh sample, a significant (*p* < 0.05) increase in level in the samples dried at 90 °C and 50 °C, although the difference between both samples was not significant (*p* > 0.05). Drying at 60 and 70 °C maintained the same level of TGC as found in the fresh sample. A slight but significant decrease was observed only in the samples dried at 80 °C. During food processing or storage, the degradation of glucosinolates occurs due to both chemical reactions and thermal effects [6]. Volatile compounds such as isothiocyanates, nitriles, sulfides, and aldehydes are released [57], contributing to positive quality enhancement. Despite the observed differences in TGC of the samples during drying, the loss of glucosinolates may be considered to be low, implying a good thermal stability of this bio-compound. The variation in TGC may be due to a combined effect of enzymatic activity that may degrade the glucosinolates and better extractability of these bio-compounds [58] in the dried product that increased the level of TGC. Thermal degradation of glucosinolates in red cabbage has been reported by Oerlemans et al. [58] for temperatures between 80 and 123 °C. They conducted their assays on frozen microwaved red cabbage powder, whereby the enzyme myrosinase was inactivated, and reported differences in stability between the different types of glucosinolates (aliphatic, indoles, aromatic). A comparison of TGC in fresh and microwaved red cabbage showed, in the absence of enzymatic activity, an overall increase in the extractable levels for the eight individually analyzed glucosinolates.

### 3.3. Identification and Quantification of Phenolic Compounds 

Table 1 shows the identification and quantification of phenolic compounds that were searched in the Pubchem online database. The control corresponds to a freeze-dried fresh sample. The exact mass was calculated for each tentative compound found in negative mode using the monoisotopic mass of the compound (theoretical mass). Then, it was compared with the experimental exact mass, and the error was calculated. Due to their low concentration, some compounds could not be fragmented. The compound called “unknow” could correspond to a quercetin derivate by its fragment signal at *m*/*z* 477.0638. The identification results are in accordance with some data reported for red cabbage [59,60]. Different concentrations were observed depending on the drying temperature used. The predominate compounds were neochlorogenic acid at 50, 80, and 90 °C, quercetin 3′-glucuronide at 50 and sample control, chlorogenic acid at 50 and 80 °C and quercetin 4′-glucuronide at 50 and 60 °C. The common treatment would be drying at 50 °C, which presents the four phenolic compounds described. Chlorogenic and neochlorogenic acids are a polyphenol and are esters of caffeic acid and quinic acid and have been used in trials studying the treatment of advanced cancer and impaired glucose tolerance [61]. Similar values (38 ± 5 µg/g d.m.) have been reported for neochlorogenic acid in broccoli [62]. Quercetin is a major flavonoid that contributes to the reduced risk of cardiovascular disease associated with dietary ingestion of fruits and vegetables. Quercetin derivates have been found in ethanolic extract of broccoli in a concentration range between 0.30 and 26.5 mg/g d.m.). Other compounds found were ferulic acid, caffeic acid, sinapic acid, and rutin, which coincided with other results found in the literature [62].

### 3.4. Amino Acids and Fatty Acids Profiles

Amino acids (AAs) are classified as essential or nonessential according to their regulatory functions in cells and roles in nutrition and body homeostasis [63]. Thermal treatment may improve, reduce, or maintain the essential AA content depending on the drying conditions or the amino acid itself [64]. Progress of proteolysis during the drying process has also been reported and usually causes a decrease in amino acid content [65]. However, during the drying of the red cabbage sample, a rather significant increase was observed, indicating an improvement [64]. The amino acids profile of red cabbage after drying at air temperature between 50 and 90 °C is shown in Table 2. The content of amino acids was determined in dry matter, and altogether, 13 of them were identified. Tryptophane was not determined. Among the amino acids found in the red cabbage samples, six of the nine essential amino acids, namely isoleucine, phenylalanine, threonine, lysine, valine, and leucine, were present. A positive change in the amino acid profile was observed in the samples dried at air temperature above 70 °C, being significantly higher than amino acid contents in the samples dried at 50 and 60 °C. In general, a significant increase (*p* < 0.05) in the individual amino acid was observed, except for tyrosine, which showed slight and almost non-significant changes with temperature. The individual amino acid contents in the samples dried at 70, 80, and 90 °C can be classified into three homogeneous groups with significant differences (*p* < 0.05). However, the differences could be considered in the order of their magnitude not relevant. The same can be said for the amino acid contents of the samples dried at 50 and 60 °C, where the difference between each other displayed a similar behavior. The two predominant amino acids in the red cabbage samples were glutamic acid and arginine, and a noticeable increase was observed in the samples dried at temperatures above 70 °C. While glutamic acid is nonessential, arginine is considered semi-essential. Nonetheless, glutamic acid is an important neurotransmitter that has a critical role in synaptic nerves’ function and maintenance, being also a component of memory. Arginine, on the other hand, is a functional amino acid known to regulate key metabolic pathways required for maintenance, growth, reproduction, and immunity [63].

The fatty acid profiles of red cabbage dried at temperatures between 50 and 90 °C are shown in Table 3. Eight saturated fatty acids (SFA), six monounsaturated fatty acids (MFA), and five polyunsaturated fatty acids were determined. In general, the contents of each individual fatty acid determined in the samples of red cabbage were hardly affected by drying temperatures. Only slight differences were observed, showing relatively good thermal stability of the fatty acids in the investigated temperature range. Short-chain fatty acids were not found in the red cabbage samples. Mid-chain saturated fatty acids, C12-0 (lauric acid) and C14-0 (myristic acid), were not detected. Only an odd-chain fatty acid C15-0 (pentadecanoic acid) was detected, along with the saturated and the monounsaturated long-odd-chain fatty acids (heptadecanoic acid) C17:0 and C17:1. Otherwise, long-chain (C16:0 to C18:0) and very long-chain (C20:0 and C22:0) saturated fatty acids, as well as monounsaturated and polyunsaturated long-chain and very long-chain fatty acids compose the fatty profile (Table 3) of red cabbage. The heptadecanoic acid (margaric acid) in the saturated and monounsaturated form was detected in a small but relevant quantity in red cabbage. It is a rare fatty acid, but its occurrence in some varieties of olive oils [66] or in seed oil of Portia tree (*Thespesia populnea*) has been reported [67]. Among the saturated fatty acids, palmitic acid (C16:0) was predominant, followed by stearic acid (C18:0). Palmitic acid as a saturated fatty acid is related to an increased level of low-density lipoprotein (LDL) and total cholesterol. However, the consumption of red cabbage should not exceed 10% of total energy intake, which is recommended as a maximum limit by the joint WHO/FAO expert consultation [68], so that its presence in red cabbage is not a health hazard. On the other hand, it has been reported that diets rich in PUFAs would be associated with a lower risk of developing PD [69]. In this sense, the polyunsaturated linoleic acid (C18:2n6c) and especially α-linolenic acid (C18:3n3) occurred as the main fatty acids and are both essential fatty acids [70]. These acids have been reported to have anti-inflammatory potential in microglial cells [71] and to improve cognitive dysfunction [72], which would give them a neuroprotective characteristic. Moreover, consumption of α-linolenic acid has been reported to be associated with a moderately lower risk of cardiovascular disease [70]. Dietary intake of α-linolenic acid was also found to improve lipid profiles by decreasing triglycerides and total cholesterol, especially low-density lipoprotein [73]. The occurrence of these omega-3 fatty acids in red cabbage makes it, therefore, more affordable and readily available. 

### 3.5. Antioxidant Activities

**Figure 3:**
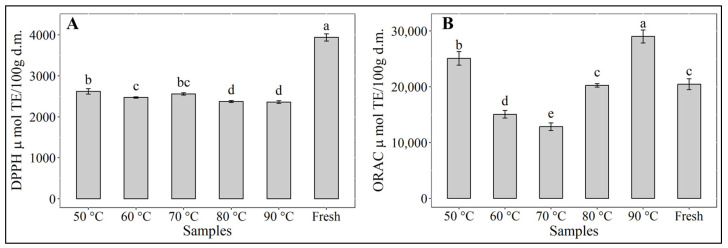
Antioxidant potential of red cabbage extracts. (**A**) DPPH assay and (**B**) ORAC assay. Different letters indicate significant differences (*p* > 0.05).

The antioxidant activity of the hot-air-dried samples of red cabbage can be seen in Figure 3. The antioxidant assays used to verify the effect of drying temperature showed red cabbage to be quite resilient to heat treatment, with drying time being hardly relevant in the investigated temperature range. Only around 40% loss of antioxidant activity (DPPH) was observed, which is comparable to values reported by López et al. [74] for vacuum drying of murta berries. In this study, the results of DPPH and ORAC assays showed marked differences, as expected, due to the different oxidation reaction mechanisms of either method [75]. The level of antioxidant activity in both cases remained high after drying at any of the selected experimental drying temperatures, although in the case of the DPPH assays, a significant (*p* < 0.05) drop in antioxidant capacity was observed compared to that of the fresh sample, similar to the report from Xu et al. [76] for white cabbage. Significant differences (*p* < 0.05) were observed between samples dried at 80 and 90 °C and those dried at 50, 60 and 70 °C. In the case of the ORAC assays, antioxidant activity showed significant differences (*p* < 0.05) at any of the drying temperatures, with the highest and the second highest values at 90 and 50 °C, respectively, being both values greater than the antioxidant activity of the fresh sample. Compared to the fresh sample, antioxidant activity at 60 and 70 °C decreased, while at 80 °C, no change was observed. This behavior could be related to the formation of flavones, flavonols, and flavonoid polymers, as reported by Liu et al. [43]. Consequently, the presence of antioxidants in dried red cabbage contributes to enhanced quality with respect to nutritional aspects. Oxidative stress has been considered as one of the main factors promoting the development and progression of PD at the cellular level [77]. Antioxidants are known for their ability to protect from oxidative cell damage, being substances that retard, impede, or discard oxidative injuries to biological macromolecules [78]. For this reason, the antioxidant activity generated by phytochemicals such as phenols, flavonoids, and anthocyanins from the diet acquire great importance since they have been related to the prevention or low risk of developing PD in humans [79]. 

### 3.6. Neuroprotective Potential

**Figure 4:**
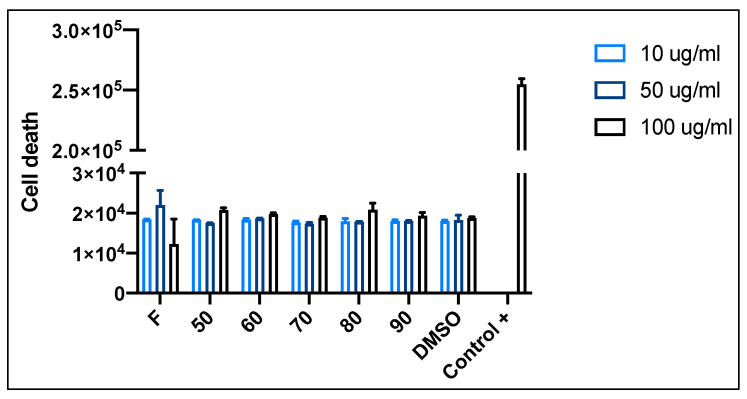
Cytotoxicity of fresh (F) and dried red cabbage extracts at different temperatures (50, 60, 70, 80 and 90 °C). Data are presented as mean and SEM of three independent experiments performed in triplicate. Statistically significant differences were detected by ordinary one-way ANOVA.

To evaluate the possible toxic effect of red cabbage extracts, cell viability assays on human neuroblastoma (SH-SY5Y) cells were performed. Figure 4 shows the cytotoxicity level of red cabbage extracts at different concentrations (10, 50, and 100 µg/mL) on SH-SY5Y cells. The permeability of the fluorescent Sytox green probe was measured after 24 h of treatment. The negative control corresponds to DMSO, and the positive control corresponds to Triton X100. According to the assays’ results, no significant difference between groups was observed. Thus, no toxic effects of the extract at any of the three concentrations used were observed on the cells. Therefore, the highest concentration (100 µg/mL) was selected to continue with the neuroprotection assay.

**Figure 5:**
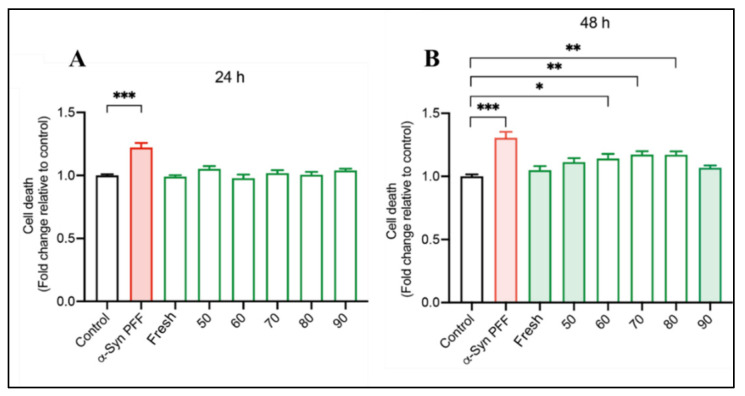
Neuroprotective effects of extracts of red cabbage. (**A**) at 24 h and (**B**) at 48 h. Data are presented as mean and SEM of three independent experiments performed in triplicate. Statistically significant differences were detected by ordinary one-way ANOVA (***: *p* < 0.001; **: *p* < 0.01; *: *p* < 0.05).

SH-SY5Y cells were treated with preformed alpha-synuclein fibrils (α-syn PFF) together with the extracts of the fresh (F) and dried red cabbage (50, 60, 70, 80, 90 °C) for 24 h (Figure 5A) and 48 h (Figure 5B). DMSO was used as a control. After treatment, the cell viability was determined using a Sytox green probe. It was observed that after 24 h of treatment, all the extracts from the fresh and the dried red cabbage samples prevented the neurotoxicity triggered by α-syn PFF. However, after 48 h, this phenomenon was observed only for the extracts from the fresh red cabbage and samples of the same, dried at 50 and 90 °C, where the neuroprotective effect was maintained. These findings show congruency with the presence and retention of some bio-compounds found in the fresh and dried red cabbage extracts, which could be associated with a neuroprotective effect. However, this property is not readily attributable to a particular type of bio-compound, considering that during drying at 50 °C and 90 °C, a high retention of TPC and TFC was observed. On the other hand, fresh red cabbage extract is a rich source of anthocyanins. Some authors have reported that bio-compounds like polyphenols, flavonoids, and anthocyanins from the natural food matrix, such as grape [80], green tea [81], mulberry [82], *morus alba* fruit [83], mandarin juice [84], elderberry [85], Brazilian green propolis [86], among others, have a neuroprotective effect associated to protection against oxidative stress or neuroinflammation. The specific mechanism of action behind the neuroprotective effect of the bio-compounds in red cabbage extracts is not easy to determine. However, it has been reported that anthocyanins present in black carrot extracts act by inhibiting ROS-mediated oxidative stress and apoptosis [87], while flavonoids in mandarin juice extracts generate protection against the overproduction of ROS in mitochondria, nucleus, and cytoplasm of the cell, restoring the gene expression of factors linked to mitochondrial functionality [84]. Moreover, phenolic compounds such as quercetin have been reported to decrease apoptosis in neurotoxicity-induced models by modulating the autophagic pathway [88]. Therefore, these bio-compounds from red cabbage could be involved in the observed neuroprotective effect, and they would be promising molecules for future studies on alternative treatment or prevention of neurodegenerative diseases. 

### 3.7. Principal Components Analysis (PCA)

The correlation matrix of all variables is shown in Table 4, where the antioxidant capacity measured by DPPH is strongly related to TAC concentration. On the other hand, ORAC assay is positively related to TPC, TAC, and especially TFC and TGC. The antioxidant effect of TGC has been reported by some authors, who found that, in general, glucosinolates do not act as peroxyl radical scavengers and chain-breaking antioxidants. Only a few of them possess an antioxidant capacity, and this property is quite specific and moderate [89]. In this sense, Cabello-Hurtado et al. [90] showed that glucobrassicin present in cauliflower is the main glucosinolate with antioxidant effect as determined by ORAC assay, although TGC represented only a small fraction of the antioxidant activity. The synergy of TGC with various other molecules is probably more important for antioxidant properties and bio-compounds retention observed in red cabbage extracts. However, further studies are required to validate this association. Table 4 also shows the estimated values of the principal components analysis (PCA). PCA is one of the most commonly used multivariate statistical methods that reduces the dimensionality in data sets and projects them into a reduced space [91]. The first two principal components, PC_1_ and PC_2_, describe the variation among different attributes in about 77.4% with variance values of 2.89 and 1.76, respectively. PC_1_ clearly describes the variation of DPPH and TAC, while PC_2_ explains the antioxidant activity, measured by ORAC assays, related to TFC and TGC.

**Figure 6:**
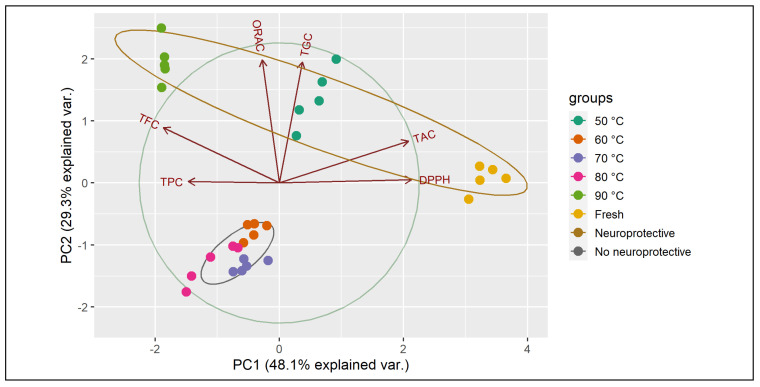
PCA assay for all red cabbage extracts related to TPC (total phenolic content), TFC (total flavonoid content), TAC (total anthocyanin content), TGC (total glucosinolate content), ORAC and DPPH (quintupled results) grouped according to neuroprotective effect at 48 h.

Figure 6 shows the PCA grouping of the neuroprotective effect at 48 h. The graph describes the relationship between the bio-compounds with the antioxidant activity and also how TAC, TFC, and TGC could be closely related to the displayed neuroprotective activity, whereby the extracts of fresh and dehydrated cabbage at 50 and 90 °C are the ones that presented greater retention of these bio-compounds. 

## 4. Conclusions

The content of bio-compounds, antioxidant activity, and neuroprotective effects of dehydrated red cabbage were affected by drying temperatures. Red cabbage has a wide variety of bio-compounds, and drying conditions affect their contents. Less exposure to heat, both in time and temperature, allows a higher retention of bio-compounds, as is the case of drying at 50 and 90 °C. It also has an effect on the antioxidant activity of the dehydrated red cabbage samples. The amino acids and fatty acids profiles confirmed red cabbage as a healthy food that is readily available. Fresh and dehydrated red cabbage extracts did not show any toxic effect and prevented the neurotoxicity triggered by α-syn PFF at 24 h treatment. Moreover, the fresh, 50 °C and 90 °C extracts maintained this effect at 48 h treatment. PCA validated the relationship between the content of bio-compounds (TAC, TFC, and TGC) and the observed neuroprotective activity. The red cabbage extracts used in assays on the cellular model of Parkinson’s disease showed a significant effect on cytotoxicity triggered by α-synuclein accumulation. These results suggest that this natural product is a potential therapeutic target to prevent or delay Parkinson’s disease progression. However, this should be validated in future clinical trials. Therefore, the present findings may promote the use of red cabbage in future studies related to PD and show its value as an affordable, functional food ingredient.

## Figures and Tables

**Table 1:** Identification of phenolic compounds in red cabbage by LC–HRMS data and concentration of phenolic compound detected.

Peak	[M − H]^−^ Theoretical Mass	[M − H]^−^ Experimental Mass	Error ppm	MS/MS (*m*/*z*)	Tentative Identification		Abbreviation
1	353.0878	353.0880	+0.55	191.0553–135.0440–179.0341	Neochlorogenic acid		NChla
2	477.0674	477.0642	−6.92	259.0129–96.9588	Quercetin 3′-glucuronide		Q3G
3	193.0506	193.0499	−3.62		Ferulic acid		FA
4	353.0878	353.0879	+0.28	135.0441–191.0552–173.0445	Chlorogenic acid		Chla
5	unknown	535.0223		336.9931–477.0638	Quercetin unknown derivate		QUd
6	179.0350	179.0341	−5.03	135.0443	Caffeic acid		CA
7	477.0675	477.0645	−6.21	468.0269–96.9588	Quercetin 4’-glucuronide		Q4G
8	223.0612	223.0608	−1.79		Sinapic acid		SA
9	609.1461	609.1421	−6.56		Rutin		R
**Tentative** **Compound**		**50 °C**	**60 °C**	**70 °C**	**80 °C**	**90 °C**	**Control**
NChla		24.54 ± 3.69 ^ab^	0.63 ± 0.11 ^d^	8.83 ± 0.11 ^c^	30.78 ± 5.57 ^a^	24.04 ± 1.26 ^ab^	19.47 ± 1.83 ^b^
Q3G		16.09 ± 0.27 ^b^	14.36 ± 3.15 ^b^	12.81 ± 0.34 ^bc^	9.07 ± 2.84 ^c^	12.76 ± 0.06 ^bc^	26.52 ± 1.92 ^a^
FA		<LOQ	<LOQ	ND	<LOQ	<LOQ	ND
Chla		11.44 ± 0.96 ^a^	0.27 ± 0.05 ^b^	ND	12.51 ± 6.19 ^a^	ND	ND
QUd		1.43 ± 0.10 ^a^	0.99 ± 0.53 ^abc^	0.44 ± 0.02 ^bc^	0.47 ± 0.41 ^bc^	0.30 ± 0.03 ^c^	1.12 ±0.01 ^ab^
CA		1.23 ± 0.06 ^b^	0.48 ± 0.07 ^c^	0.95 ± 0.09 ^b^	2.29 ± 0.35 ^a^	2.13 ± 0.06 ^a^	1.27 ± 0.04 ^b^
Q4Ge		11.73 ± 1.33 ^a^	12.13 ± 0.62 ^a^	ND	3.40 ± 2.26 ^b^	ND	ND
SA		0.43 ± 0.10 ^c^	<LOQ	<LOQ	1.60 ± 0.03 ^a^	0.77 ± 0.03 ^b^	<LOQ
R		0.69 ± 0.18 ^d^	0.90 ± 0.17 ^cd^	1.62 ± 0.02 ^a^	0.76 ± 0.24 ^d^	1.22 ± 0.10 ^bc^	1.33 ± 0.06 ^ab^

Results were expressed as mean ± standard deviation (sd) in µg/g d.m. (n = 2). ND: not detected. <LOQ: Lower Limit of Quantification (LOQ: 0.23 μg/g, S/N > 3). Values in the same row with different superscript letters indicate significant differences (*p* < 0.05).

**Table 2:** Amino acids content of dried red cabbage at different temperatures.

Amino Acids g/100 g d.m.	Drying Temperature (°C)				
	50	60	70	80	90
ASP	0.93 ± 0.06 ^c^	0.60 ± 0.27 ^c^	1.81 ± 0.33 ^b^	2.68 ± 0.45 ^a^	2.06 ± 0.36 ^ab^
GLU	2.26 ± 0.14 ^cd^	1.25 ± 0.50 ^d^	3.87 ± 0.69 ^bc^	6.42 ± 1.17 ^a^	4.61 ± 0.59 ^ab^
SER	0.50 ± 0.10 ^cd^	0.35 ± 0.15 ^d^	0.97 ± 0.20 ^bc^	1.54 ± 0.28 ^a^	1.25 ± 0.19 ^ab^
GLY	0.35 ± 0.19 ^a^	0.39 ± 0.08 ^a^	0.65 ± 0.42 ^a^	0.94 ± 0.26 ^a^	0.72 ± 0.04 ^a^
THR	ND	ND	0.69 ± 0.57 ^a^	1.35 ± 0.22 ^a^	1.02 ± 0.11 ^a^
ARG	0.61 ± 0.31 ^c^	0.23 ± 0.08 ^c^	3.04 ± 0.24 ^b^	3.75 ± 0.54 ^ab^	3.90 ± 0.09 ^a^
ALA	0.32 ± 0.04 ^b^	0.25 ± 0.11 ^b^	0.52 ± 0.11 ^b^	1.14 ± 0.23 ^a^	1.31 ± 0.06 ^a^
TYR	0.67 ± 0.23 ^abc^	0.44 ± 0.15 ^bc^	0.29 ± 0.15 ^c^	0.93 ± 0.08 ^a^	0.86 ± 0.15 ^ab^
VAL	0.42 ± 0.09 ^bc^	0.25 ± 0.08 ^c^	0.71 ± 0.13 ^ab^	1.06 ± 0.24 ^a^	0.86 ± 0.12 ^a^
PHE	0.36 ± 0.00 ^b^	0.35 ± 0.18 ^b^	0.61 ± 0.09 ^b^	1.81 ± 0.42 ^a^	1.38 ± 0.17 ^a^
ILE	0.71 ± 0.24 ^bc^	0.39 ± 0.23 ^c^	1.38 ± 0.31 ^ab^	2.05 ± 0.36 ^a^	1.44 ± 0.07 ^a^
LEU	0.05 ± 0.05 ^b^	ND	0.36 ± 0.05 ^a^	0.45 ± 0.15 ^a^	0.35 ± 0.05 ^a^
LYS	0.39 ± 0.10 ^b^	0.29 ± 0.19 ^b^	0.91 ± 0.15 ^a^	1.32 ± 0.18 ^a^	1.04 ± 0.18 ^a^

Values are expressed as mean ± sd of three replicated determinations. Values in the same row with different superscript letters indicate significant differences (*p* < 0.05). ND: No detected.

**Table 3:** Fatty acid profiles of dried red cabbage at different temperatures.

g/100 g FAMES	Drying Temperature (°C)				
	50	60	70	80	90
SFA					
C12:0	ND	0.26 ± 0.08 ^a^	0.12 ± 0.00 ^b^	0.1 ± 0.01 ^b^	0.12 ± 0.01 ^b^
C14:0	ND	0.34 ± 0.02 ^a^	0.23 ± 0.01 ^b^	0.19 ± 0.00 ^b^	0.2 ± 0.01 ^b^
C15:0	0.5 ± 0.07 ^a^	0.58 ± 0.09 ^a^	0.55 ± 0.04 ^a^	0.45 ± 0.03 ^a^	0.51 ± 0.01 ^a^
C16:0	18.86 ± 0.88 ^a^	19.52 ± 1.43 ^a^	17.47 ± 0.42 ^a^	20.02 ± 0.85 ^a^	17.43 ± 0.21 ^a^
C17:0	0.87 ± 0.10 ^ab^	0.52 ± 0.08 ^c^	0.71 ± 0.07 ^bc^	0.98 ± 0.07 ^a^	0.99 ± 0.04 ^a^
C18:0	4.16 ± 0.10 ^b^	5.95 ± 0.97 ^a^	3.97 ± 0.05 ^b^	3.14 ± 0.15 ^b^	3.63 ± 0.05 ^b^
C20:0	0.5 ± 0.07 ^a^	0.81 ± 0.19 ^a^	0.77 ± 0.03 ^a^	0.56 ± 0.10 ^a^	0.74 ± 0.01 ^a^
C22:0	0.31 ± 0.00 ^a^	0.54 ± 0.18 ^a^	0.39 ± 0.04 ^a^	0.4 ± 0.04 ^a^	0.44 ± 0.08 ^a^
MFA					
C16:1	0.32 ± 0.09 ^a^	0.41 ± 0.14 ^a^	0.32 ± 0.05 ^a^	0.32 ± 0.03 ^a^	0.42 ± 0.02 ^a^
C17:1	0.24 ± 0.08 ^a^	0.35 ± 0.13 ^a^	0.3 ± 0.06 ^a^	0.2 ± 0.09 ^a^	0.26 ± 0.01 ^a^
C18:1n9c/C18:1n9t	1.42 ± 0.39 ^b^	2.26 ± 0.33 ^a^	1.77 ± 0.21 ^ab^	0.7 ± 0.26 ^b^	1.07 ± 0.06 ^b^
C20:1n9	0.11 ± 0.04 ^a^	0.34 ± 0.13 ^a^	0.21 ± 0.03 ^a^	0.11 ± 0.03 ^a^	ND
C22:1n9	ND	0.11 ± 0.00 ^a^	0.06 ± 0.04 ^a^	0.06 ± 0.04 ^a^	0.09 ± 0.05 ^a^
C24:1n9	0.51 ± 0.04 ^a^	0.78 ± 0.30 ^a^	0.75 ± 0.22 ^a^	0.56 ± 0.04 ^a^	0.42 ± 0.20 ^a^
PFA					
C18:2n6c	20.7 ± 0.39 ^ab^	19.38 ± 2.40 ^ab^	22.36 ± 0.14 ^a^	17.34 ± 0.18 ^b^	18.53 ± 0.12 ^b^
C18:3n3	51.19 ± 1.09 ^a^	48.39 ± 1.08 ^ab^	48.76 ± 0.36 ^ab^	54.17 ± 0.43 ^a^	54.31 ± 0.42 ^a^
C20:2	0.13 ± 0.05 ^a^	0.2 ± 0.03 ^a^	0.23 ± 0.03 ^a^	0.19 ± 0.05 ^a^	0.19 ± 0.03 ^a^
C20:3n3	0.11 ± 0.03 ^b^	0.31 ± 0.10 ^a^	0.2 ± 0.02 ^ab^	0.2 ± 0.06 ^ab^	0.19 ± 0.03 ^ab^
C22:6n3	0.27 ± 0.00 ^a^	0.65 ± 0.24 ^a^	0.81 ± 0.42 ^a^	0.39 ± 0.11 ^a^	0.48 ± 0.25 ^a^
Total SFA	24.99 ± 0.87 ^a^	28.52 ± 3.04 ^a^	24.22 ± 0.34 ^a^	25.84 ± 0.57 ^a^	24.07 ± 0.24 ^a^
Total MFA	2.61 ± 0.55 ^b^	4.25 ± 1.03 ^a^	3.41 ± 0.18 ^ab^	1.91 ± 0.36 ^b^	2.23 ± 0.16 ^b^
Total PFA	72.39 ± 1.41 ^ab^	68.93 ± 3.85 ^b^	72.36 ± 0.40 ^ab^	72.28 ± 0.40 ^ab^	73.7 ± 0.35 ^a^

Values are expressed as mean ± sd of three replicated determinations. Values in the same raw with different superscript letters indicate significant differences (*p* < 0.05). FAMES (Fatty Acid Methyl Ester Standard), SFA (Saturated fatty acids), MFA (Monounsaturated fatty acids), and PFA (Polyunsaturated fatty acids). ND: No detected.

**Table 4:** Correlation between the variables and estimates for PC_1_ and PC_2_.

	ORAC	DPPH	TPC	TFC	TGC	TAC
ORAC	1					
DPPH	−0.04512324	1				
TPC	0.1454054	−0.38457781	1			
TFC	0.38914863	−0.88279979	0.27005582	1		
TGC	0.54154351	0.18675585	−0.02073628	0.17229989	1	
TAC	0.15248469	0.82962777	−0.67074275	−0.57943842	0.35656316	1
	PC_1_			PC_2_		
ORAC	−0.07164693			0.66194871		
DPPH	0.55538337			0.01775146		
TPC	−0.38219393			0.00680021		
TFC	−0.4869898			0.29703139		
TGC	0.09657944			0.65004107		
TAC	0.54208594			0.22512553		

TPC (total phenolic content), TFC (total flavonoid content), TAC (total anthocyanin content) and TGC (total glucosinolate content).

## Data Availability

Data is contained within the article.
